# Progress and Future Directions of the NCAA-DoD Concussion Assessment, Research, and Education (CARE) Consortium and Mind Matters Challenge at the US Service Academies

**DOI:** 10.3389/fneur.2020.542733

**Published:** 2020-09-24

**Authors:** Megan N. Houston, Kevin J. O'Donovan, Jesse R. Trump, Rachel M. Brodeur, Gerald T. McGinty, J. Kenneth Wickiser, Christopher J. D'Lauro, Jonathan C. Jackson, Steven J. Svoboda, Adam J. Susmarski, Steven P. Broglio, Thomas W. McAllister, Michael A. McCrea, Paul Pasquina, Kenneth L. Cameron

**Affiliations:** ^1^Department of Orthopaedic Research, John A. Feagin Jr. Sports Medicine Fellowship, Keller Army Community Hospital, West Point, NY, United States; ^2^Department of Chemistry and Life Sciences, United States Military Academy, West Point, NY, United States; ^3^United States Coast Guard Academy, New London, CT, United States; ^4^United States Air Force Academy, Colorado Springs, CO, United States; ^5^Medstar Orthopaedic Institute, Washington, DC, United States; ^6^United States Naval Academy, Annapolis, MD, United States; ^7^Michigan Concussion Center, University of Michigan, Ann Arbor, MI, United States; ^8^Department of Psychiatry, Indiana University School of Medicine, Indianapolis, IN, United States; ^9^Department of Neurosurgery, Medical College of Wisconsin, Milwaukee, WI, United States; ^10^Department of Physical Medicine and Rehabilitation, Uniformed Services University of the Health Sciences, Bethesda, MD, United States

**Keywords:** concussion, mild traumatic brain injury, biomarkers, baseline, head impact exposure

## Abstract

Despite the significant impact that concussion has on military service members, significant gaps remain in our understanding of the optimal diagnostic, management, and return to activity/duty criteria to mitigate the consequences of concussion. In response to these significant knowledge gaps, the US Department of Defense (DoD) and the National Collegiate Athletic Association (NCAA) partnered to form the NCAA-DoD Grand Alliance in 2014. The NCAA-DoD CARE Consortium was established with the aim of creating a national multisite research network to study the clinical and neurobiological natural history of concussion in NCAA athletes and military Service Academy cadets and midshipmen. In addition to the data collected for the larger CARE Consortium effort, the service academies have pursued military-specific lines of research relevant to operational and medical readiness associated with concussion. The purpose of this article is to describe the structure of the NCAA-DoD Grand Alliance efforts at the service academies, as well as discuss military-specific research objectives and provide an overview of progress to date. A secondary objective is to discuss the challenges associated with conducting large-scale studies in the Service Academy environment and highlight future directions for concussion research endeavors across the CARE Service Academy sites.

## Introduction

Globally, an estimated 64 million to 74 million new cases of traumatic brain injury (TBI) occur each year (1). The vast majority of these cases are classified as mild TBI (mTBI) and are more commonly referred to as *concussion*. According to consensus guidelines, concussion is defined as “a change in brain function following a force to the head, which may be accompanied by temporary loss of consciousness, but is identified in awake individuals with measures of neurologic and cognitive dysfunction” (2). The consequences of concussion vary but often include cognitive impairment, physical symptoms, and emotional and somatic changes (3). Although most concussion symptoms and deficits resolve within days to weeks, there has recently been growing concern about the potential long-term consequences of injury (4, 5). Despite these recent concerns, concussions and concussion sequelae are not new (6). Public concerns date back to the booming industrial world that transformed the risk of head injury in labor and leisure (7, 8). Over the past 20 years, fears heightened as soldiers returned home from war with blast injuries and sports such as football and boxing gained popularity (9). These concerns prompted further investigation into concussion and stimulated numerous studies that would transform our knowledge and understanding of what has been termed the *invisible wound* in military service members (10). Since 2000, more than 342,000 concussions have been diagnosed in US service members (11). While more severe blast injuries have received a significant amount of attention within military populations, the majority of mTBIs and concussions in this population are not related to combat or blast injury and occur within the continental United States (12). Even with the increased attention concussion has received and the immense progress that has been made in the field, the natural history and clinical management of concussion remain challenging, and the consequences of repetitive head impact exposure over time are not well-understood.

Each year, 15,000–32,000 service members sustain concussions from trauma related to sports, military training accidents, motor vehicle accidents, and combat (11, 12). Beyond the acute physical effects of concussion, which include microvascular damage, neuroinflammation, elevations in phosphorylated tau, and multifocal axonal injury, emerging evidence suggests that a small proportion of individuals may develop persistent cognitive and behavioral changes after mild neurotrauma (13). Despite the impact that concussion has on military service members, significant gaps remain in our understanding of the optimal diagnostic, management, and return to activity/duty criteria to mitigate the consequences of concussion. Specifically, there is insufficient evidence supporting the objectivity (i.e., sensitivity and specificity) of our assessment measures, the optimal treatment strategies to mitigate the impact of concussion, and our ability to predict longer-term outcomes following injury. Additionally, further exploration into the overlap between concussion and post-traumatic stress disorder (PTSD) is warranted. Within the military population, concussion and PTSD frequently coexist as both often occur during a traumatic experience (14). The overlap in concussion and PTSD mechanisms and symptoms presents challenges to differential diagnosis and treatment (14).

**Figure 1:**
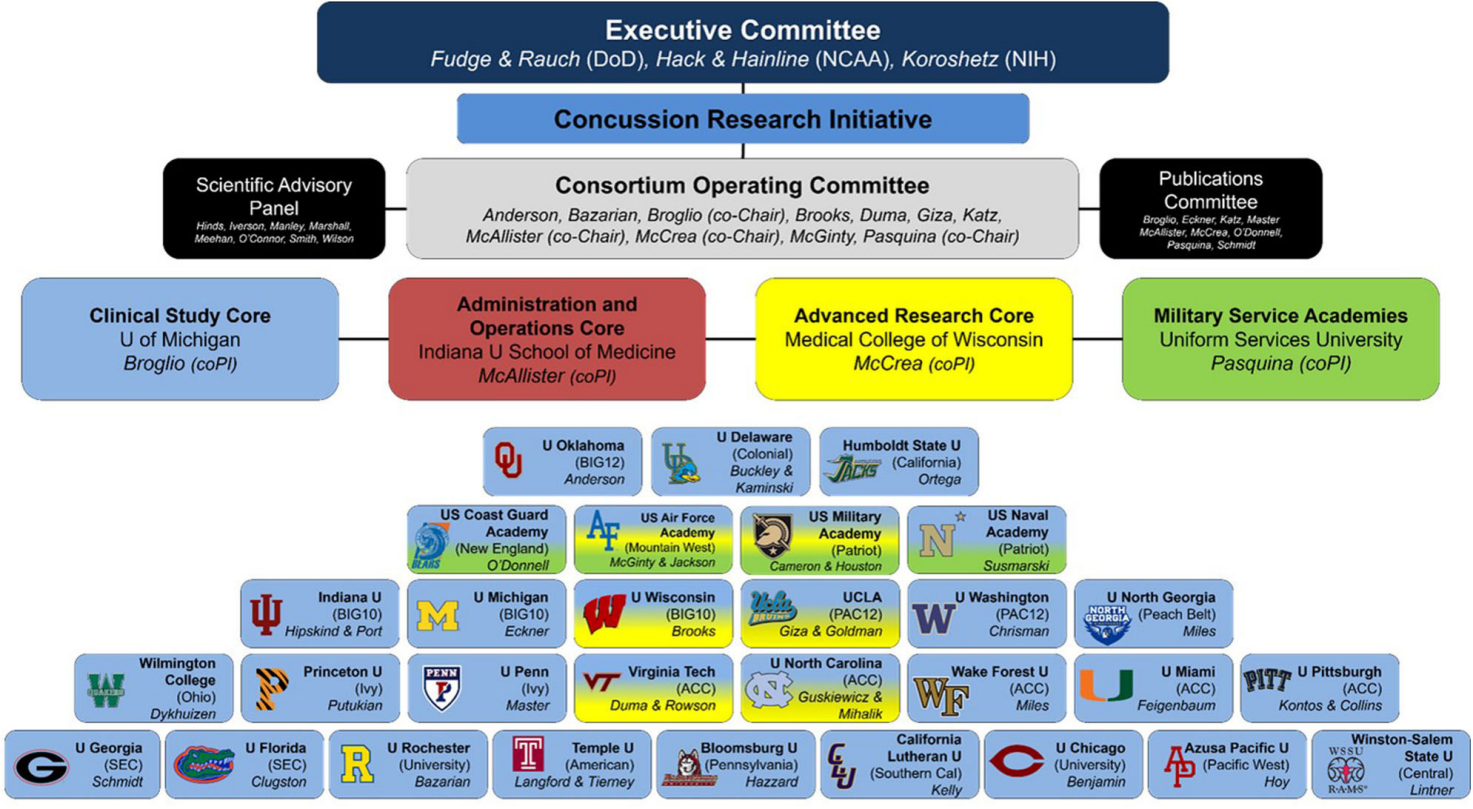
CARE Consortium structure as of February 2020. Modified with permission from: Broglio et al. (15).

**Figure 2:**
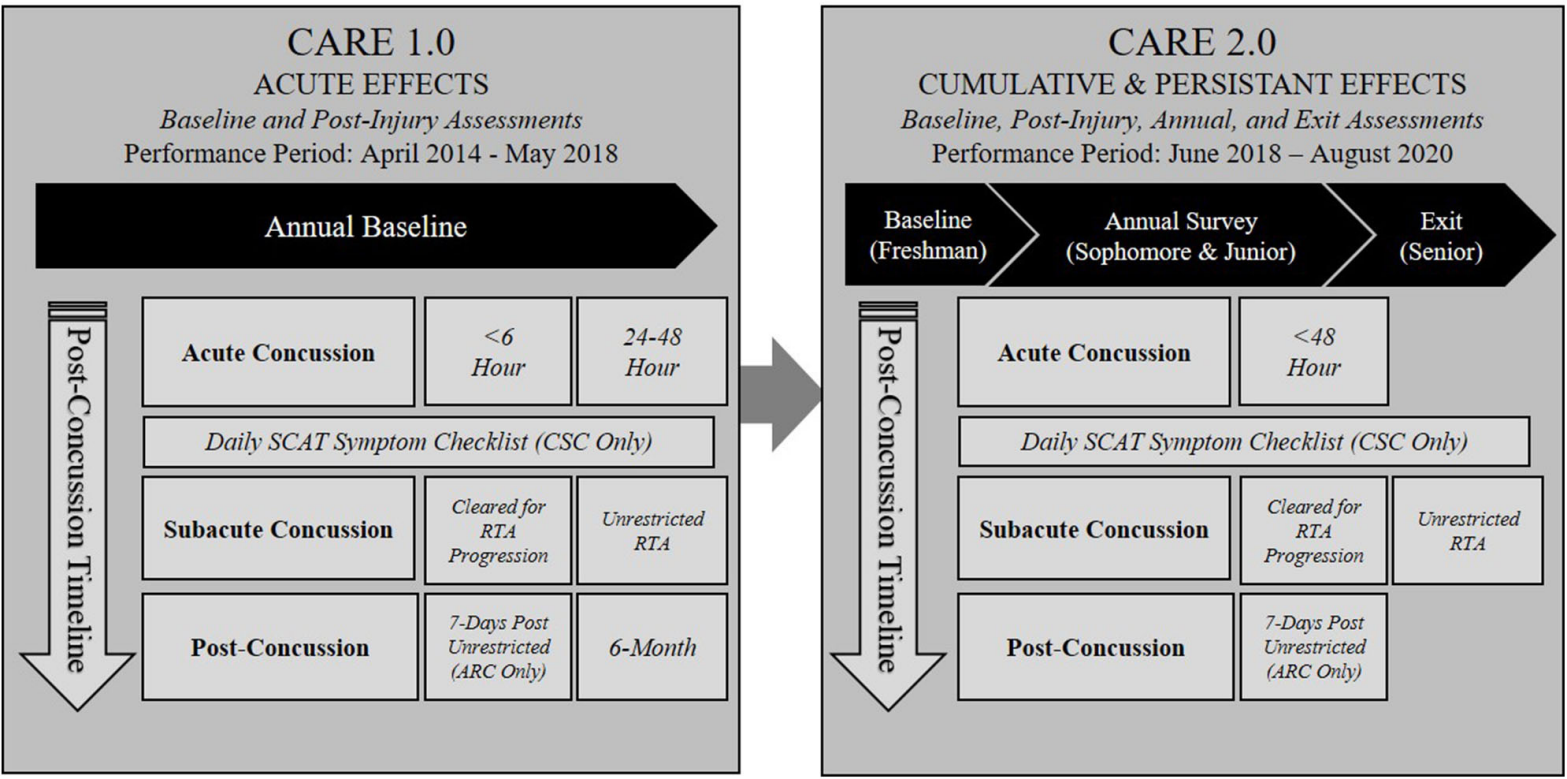
CARE Service Academy phases and collection timelines. ARC, Advanced Research Core; CSC, Clinical Study Core; CARE, Concussion Assessment, Research, and Education; RTA, return to activity.

In response to these significant knowledge gaps, the US Department of Defense (DoD) and National Collegiate Athletic Association (NCAA) partnered to form the NCAA-DoD Grand Alliance in 2014. The NCAA-DoD Grand Alliance was formed with two main arms: the Mind Matters Challenge and the Concussion Research Initiative that evolved into the Concussion Assessment, Research, and Education (CARE) Consortium (15). The NCAA-DoD CARE Consortium was established with the aim of creating a national multisite research network to study the clinical and neurobiological natural history of concussion in NCAA athletes and military Service Academy cadets and midshipmen. The overall structure and function of the CARE Consortium have been described in detail elsewhere (15), but a brief overview is provided here. Over the last 6 years, 30 colleges and institutions, including four US service academies, have joined the CARE Consortium to collectively investigate the complex nature of concussion. The initial CARE Consortium structure comprised three arms, the Administration and Operations Core as in the figure. Clinical Study Core (CSC), and Advanced Research Core (ARC). Since commencement, this has expanded to include the Service Academy Longitudinal mTBI Outcomes Study (SALTOS) arm. An updated structure of the current CARE Consortium is illustrated in Figure 1. In its first phase (CARE 1.0), CARE focused on understanding the acute and subacute effects of concussion and factors that predict poor outcomes. In its second phase (CARE 2.0), the CARE Consortium has sought to further understand and characterize the intermediate-term cumulative and persistent effects of concussion(s) and/or repetitive head impact exposure on neurological health and military performance. In addition, this effort will hopefully also establish a stronger framework for conducting an even longer-term prospective study. The CARE Consortium phases and collection timelines are outlined in Figure 2. Recognizing the unique demands of military service members and the commonalties between collegiate athletes and tactical athletes (e.g., age, physical demands, operating as teams), the four service academies expanded CARE Consortium enrollment to cadets and midshipmen not participating in NCAA athletics. This decision was made because these individuals may be more representative of the concussions experienced in military service members during training and other activities. In addition to the data collected for the larger CARE Consortium effort, each academy has pursued military-specific lines of research relevant to operational and medical readiness associated with concussion. The purpose of this article is to describe the structure of the NCAA-DoD Grand Alliance efforts at the service academies, as well as discuss military-specific research objectives and provide an overview of progress to date. A secondary objective is to discuss the challenges associated with conducting large-scale studies in the Service Academy environment and highlight future directions for concussion research endeavors across the CARE Service Academy sites.

## Structure of the Grand Alliance at the us Service Academies

The US Air Force (USAFA), Coast Guard (USCGA), Military (USMA), and Naval (USNA) Academies initially joined the NCAA-DoD Grand Alliance to enroll their NCAA athletes; however, in a concerted effort to expand the breadth of the study, the academies began enrolling non-NCAA athlete cadets and midshipmen. As part of the Grand Alliance, the service academies have participated in the CARE Consortium's CSC and ARC and the Educational Mind Matters Challenge. All four service academies initially joined the alliance as part of the CARE Consortium's CSC arm. In 2016, given the enrollment success at the academies and the ability of individual sites to execute aspects of the advanced arm, USAFA and USMA were asked to join the ARC, in addition to participation in the CSC. As part of the ARC, both sites incorporated blood biomarker collections into their baseline and post-injury protocols and instrumented select cadet athletes with head impact measurement technology to assess head impact exposure and concussion biomechanics. Additionally, investigators at USAFA and USMA have been executing educational research projects as part of the Mind Matters Challenge. As of December 2019, the service academies have collectively enrolled roughly 36% of the CARE Consortium's CSC participants (16,372 of 45,239) and 50% of the ARC participants (1,303 of 2,657) and captured post-injury data on 36% of the CSC concussions (1,621 of 4,470) and 65% of the ARC concussions (394 of 604).

To successfully execute the large-scale scope of the CARE Consortium and Mind Matters activities, each academy had to develop a concept of operations that included an annual baseline assessment for all participants. USAFA, USMA, and USNA each had more than 4,000 eligible cadets and midshipmen to baseline, and the USCGA had more than 1,000 eligible cadets to baseline. The concept of operations also included a plan for researchers and clinicians to successfully capture post-injury data throughout the concussion management process. Investigators at each academy worked with their local medical staff to integrate the research efforts into current clinical standard-of-care best practices for concussion management. USAFA, USCGA, USMA, and USNA were able to implement annual concussion baseline testing for all cadets and midshipmen, so that the information collected as part of the clinical standard of care could be used for research to ensure no duplication of effort. Preinjury baseline testing is primarily conducted in the summer months with the intent of capturing the assessments prior to the academic year; however, the timing varies somewhat by site. Freshmen at USCGA and USAFA complete baseline testing in late June/early July prior to their 8-week basic cadet training. USMA freshmen complete baseline testing after cadet basic training, during the first 2 weeks of the academic year in late August, whereas USNA freshmen complete baseline testing during preparticipation sports physicals throughout the month of July. During baseline testing, cadets and midshipmen are introduced to the study and given an opportunity to consent. Only the cadets and midshipmen who consent to participate in the research have their data uploaded into the study database. Local Institutional Review Board and Human Research Protection Office approvals are in place at each site.

As CARE sites, each Service Academy is required to execute the Level A assessments at baseline and at clinically relevant time points post-concussion. As previously described (15), Level A assessments include demographics, medical history, neurocognitive assessment, neurological status, postural stability, and symptoms. The demographic and medical history data, as well as the symptom inventories, are standard at all 30 CARE sites including the Service Academies. Participants complete the information on paper or electronically in the research database. To assess neurological status and postural stability, cadets and midshipmen complete the Standardized Assessment of Concussion and Balance Error Scoring System (BESS) one-on-one with a trained test administrator. To enhance exploration of performance and predictive abilities, CARE Consortium sites were allowed to pick their own neurocognitive assessment from a predetermined list of test batteries. Cadets at USAFA, USMA, and USCGA complete the Immediate Post-Concussion Assessment and Cognitive Testing (ImPACT) and midshipmen at USNA complete the Automated Neuropsychological Assessment Metrics (ANAM). In 2018, the CARE Consortium transitioned into CARE 2.0, described in Figure 2. Within the CARE 2.0 protocol, participants no longer complete the annual baseline assessment, but rather an initial baseline upon entry to an academy. The annual baseline assessment was removed as the CARE data suggested an overlap in performance from year-to-year, signifying that baseline testing may not yield vital insights for concussion management beyond the initial evaluation in college students (16). Furthermore, to target the cumulative and persistent effects of concussion, an annual electronic survey and exit assessments prior to graduation were added. Table 1 includes the assessments and summarizes the collection timeline and transition from CARE 1.0 to CARE 2.0 at the service academies. The post-injury assessments were also adjusted. Changes included eliminating the <6-h and 6-month time points, because of a substantial amount of missing data, as it proved difficult to capture those time points. Table 2 reflects these changes and outlines the measures collected during both phases.

**Table 1:** Summary of the CARE Service Academy standard time points and assessments.

	**Baseline assessment**	**Annual online survey**	**Exit assessment**
CSC	Demographics and medical history ImPACT or ANAM Standardized Assessment of Concussion Balance Error Scoring System SCAT symptoms Brief Symptom Inventory-18 Neurobehavioral Symptom Inventory Wechsler Test of Adult Reading	Demographics and medical history Neurobehavioral Symptom Inventory	Demographics and medical history ImPACT or ANAM Standardized Assessment of Concussion Balance Error Scoring System SCAT symptoms Brief Symptom Inventory-18 Neurobehavioral Symptom Inventory
ARC	Blood biomarker collection		Blood biomarker collection
Military-relevant outcomes	Post-traumatic Stress Disorder Checklist	Disciplinary Actions Military Academic Status Physical Fitness Test Scores	Disciplinary actions Military Academic Status Physical Fitness Test Scores Post-traumatic Stress Disorder Checklist
Patient-reported outcomes		Alcohol Use Disorders Identification Test Life Change Index Scale Patient Health Questionnaire-8 Pittsburgh Sleep Quality Index Satisfaction With Life Scale	Alcohol Use Disorders Identification Test Athletic Identity Measurement Scale Connor-Davidson Resilience Scale Life Events Checklist Neuro-Quality of Life Cognitive Domain (short-form) Patient Health Questionnaire-9 Pittsburgh Sleep Quality Index Satisfaction with Life Scale Short-Form Health Survey-12

Time points shaded gray were added as part of CARE 2.0.

*ANAM, Automated Neuropsychological Assessment Metrics; ARC, Advanced Research Core; CSC, Clinical Study Core; CARE, Concussion Assessment, Research, and Education; ImPACT, Immediate Post-concussion Assessment and Cognitive Testing; SCAT, Sport Concussion Assessment Tool*.

**Table 2:** Summary of the CARE Service Academy post-concussion time points and assessments.

	**Acute concussion**		**Subacute concussion**		**Post-concussion**	
	**<6 h**	**24–48 h**	**Cleared for return to activity progression**	**Unrestricted return to activity**	**7 Days following return to activity (ARC only)**	**6-Month post-injury**
CSC	SAC BESS SCAT symptoms	ImPACT or ANAM SAC BESS SCAT symptoms BSI-18	ImPACT or ANAM SAC BESS SCAT symptoms BSI-18	ImPACT or ANAM SAC BESS SCAT symptoms BSI-18		ImPACT or ANAM SAC BESS SCAT symptoms BSI-18
ARC	Blood biomarker collection	Blood biomarker collection	Blood biomarker collection		Blood biomarker collection ImPACT SAC BESS SCAT symptoms BSI-18	Blood biomarker collection

Time points shaded gray remained in place for CARE 2.0; non-shaded time points were discontinued at the end of CARE 1.0.

*ANAM, Automated Neuropsychological Assessment Metrics; ARC, Advanced Research Core; CSC, Clinical Study Core; CARE, Concussion Assessment, Research, and Education; ImPACT, Immediate Post-concussion Assessment and Cognitive Testing; SCAT, Sport Concussion Assessment Tool*.

In an effort to track the cumulative and persistent effects of concussion and/or repetitive head impact exposure on overall health and military performance, one of the aims of CARE 2.0, the service academies began collecting patient-reported outcomes (PROs) and military-relevant outcomes (MROs). The PROs include general health and wellness outcomes that target areas such as sleep, quality of life, alcohol use, and resilience. The MROs include physical, military, and academic performance scores that are unique to the Service Academy environment. Table 1 outlines the specific PROs and MROs that are collected during annual and exit assessments from CARE Service Academy participants. All of the outcomes have been previously validated (17–25) or are standard assessments of academic performance (i.e., class rank, military grade point average) (26) or physical fitness (i.e., aerobic capacity, muscular strength) (27) at the service academies. This information will allow the investigators to better understand how these factors may influence concussion recovery in the Service Academy population. Additionally, investigators will have the ability to gain greater insight into how these items change over the course of a cadet's or midshipman's 4-year career at an academy in those who sustain an incident concussion and those who do not.

CARE 2.0 also expanded biomarker collection efforts to CSC participants. Saliva collections were initiated in CSC participants to increase the samples available to generate proteomic and genomic profiles in concussed and exposed cohorts. These efforts had previously been restricted to blood samples provided by ARC participants. The intent is to compare the proteomic and genomic profiles of participants who sustain concussions during the study period to those who do not. More specifically, three cohorts will be formed: concussed, exposed, and controls. The individuals that do not sustain a concussion will be characterized as exposed or controls. Participants in the exposed group have been exposed to repetitive head impacts (i.e., football, ice hockey), whereas participants in the control group are those with limited head impact exposure (i.e., track, rowing/crew). In 2019, USAFA, USCGA, and USMA began collecting saliva samples in their respective cohorts. The collections at the service academies will vastly expand the pool of participants beyond traditional NCAA sports to include individuals who participate in competitive club sports, such as boxing, orienteering, cycling, and martial arts, and intramural athletes who compete in sports such as flag football and ultimate Frisbee. The sample is a one-time collection that can be provided during any study visit (i.e., baseline, post-injury, exit). During a collection, the participant is instructed to spit directly into an Oragene saliva collection kit (Ottawa, Ontario, Canada) until 2.0 mL of saliva is provided. The kit is then closed and stored at room temperature. To date, the service academies have collected 111 saliva samples [40 (36%) controls, 14 (13%) exposed, and 57 (51%) concussed].

## Service Academy Specific Findings to Date

Because of the unique demands of military service, training, and the increased risk of concussion in military service members, several CARE Consortium publications and presentations have focused on the Service Academy population. For a summary of the CARE Service Academy–specific publications and presentations, see Supplementary Tables 1, 2. Investigators have focused on describing baseline characteristics and identifying factors that may influence baseline performance in this population to inform concussion assessment and management (28–30). Baseline data from more than 9,700 cadets were used to establish normative values across sex, competition level (e.g., varsity, club, intramural), and varsity contact sport level (28). Overall, sex, competition level, and contact level had little to no clinically meaningful association with baseline performance or symptom reporting (28). Reported racial identity, freshman status, and academy affiliation were the covariates most commonly influencing performance and symptom reporting at baseline (28). A separate investigation into CARE Service Academy baseline data revealed that tobacco users performed significantly worse than tobacco non-users on the impulse control section of ImPACT, reported greater ImPACT symptom severity scores, and were more likely to take risks as measured by the Brief Sensation Seeking Scale (29). Although the differences detected between tobacco users and tobacco non-users were significant, these results should be interpreted with caution, as the overall effect sizes noted were small (29). Another investigation used sport history information collected at baseline to explore the association between estimated age at first exposure to contact sports in male NCAA cadets and neurocognitive performance and symptom ratings (30). Earlier estimated age at first exposure to contact sports did not relate to neurocognitive performance or subjectively experienced symptoms at baseline in male Service Academy cadets (30).

Additional Service Academy–specific publications have focused on describing the frequency of delayed reporting (31), characterizing factors that may influence concussion risk (32), and describing concussion recovery trajectories (33, 34). In a cohort of roughly 300 concussed cadets, 51% failed to immediately report their injury (31). The data suggested that female cadets, cadets injured in a setting outside of competition (e.g., practice, free time), and highly competitive cadets (e.g., NCAA or club athletes) were almost twice as likely to delay reporting than their counterparts (31). In a sample of 738 concussed cadets, female cadets [odds ratio (OR) = 2.02] and cadets with a prior concussion (OR = 1.53) were consistently associated with increased concussion risk, regardless of injury setting (i.e., sport-related, academy training-related, free time) (32). Furthermore, freshman cadets were at an eight times (OR = 8.17; 95% CI = 5.87–11.37) greater risk of academy training-related (i.e., physical education, military training, boxing) concussion compared to non-freshman (32). In a separate analysis, this concussed cohort was also used to describe concussion recovery trajectories (33). The most significant finding was that duration of symptoms alone extends beyond the typical 14-day recovery window (35). At 14 days post-concussion, only 59% of the cohort was asymptomatic, and by 28 days, 84%. The duration of symptoms exceeded 1 month for a small portion (15%) of the cohort (33). This analysis also yielded that different intrinsic and acute injury characteristics contribute to both symptom duration and return to activity protocol durations (33). Among all concussions, male sex, varsity status, and low initial symptom burden were found to be associated with significantly shorter recovery timelines (33). However, acute symptom burden was the most consistent and robust predictor of recovery time with endorsement of more than 11 symptoms, indicating longer recovery periods (33). Return-to-play timelines in USAFA cadets (mean time of 29.4 days) have also exceeded the commonly cited recovery window of 10 to 14 days (34, 35). Similar to the findings of O'Connor et al. (33), male (24.7 days) and collegiate athlete-cadets (25.4 days) returned quicker than did female (35.5 days) and non-intercollegiate (34.7 days) cadets (34). In summary, this collection of publications represents the first set of normative values for an underrepresented military population and begins the initial phases of comprehending concussion risk, reporting, and recovery patterns in Service Academy cadets and military service members.

### Mind Matters Challenge

In addition to their work as CSC and ARC sites within the CARE Consortium, USAFA, and USMA have played an instrumental role in the Mind Matters Challenge. The Mind Matters Challenge is aimed at changing important concussion safety behaviors and focuses on changing attitudes and perceptions regarding concussion in young adults and developing effective educational programs targeting young adults to improve concussion awareness and injury reporting. USMA has collaborated with investigators at the University of North Carolina–Chapel Hill (UNC) who are working to better understand how concussion beliefs, attitudes, norms, and knowledge are associated with concussion disclosure behaviors in civilian and military Service Academy populations. The research teams at USMA and UNC have also developed and are evaluating targeted educational interventions to improve concussion injury disclosure in these populations. The data that have been analyzed and published indicate an association between high intention to disclose concussion symptoms and disclosure prevalence at the time of injury (36). The strongest factor associated with intention to disclose concussion was favorable perceived social norms (i.e., perceptions of organization, social referent expectations, and actions concerning concussion), making the social environment and chain of command key targets for concussion disclosure interventions (36). Additional data indicated that concussion education exposure (education included whether participants had been formally educated about concussions) varies among new cadets upon entry to the USMA. Furthermore, exposure to multiple sources of concussion education increased the prevalence of concussion disclosure (37). This information is being used to design and evaluate an intervention platform to address factors and behaviors concerning concussion disclosure in both NCAA athletes and military Service Academy cadets. Investigators at the USAFA received a Mind Matters Challenge grant to explore the culture surrounding concussion disclosure in the military environment and the factors that may influence concussion disclosure behaviors. Thus far, the USAFA research team has established a return-to-learn program for Service Academy cadets (38). Additional advances have been made toward understanding the culture of concussion disclosure at the USAFA and indicate that non-disclosure can develop in any population where disclosure is perceived as having undesirable consequences, such as missing practice or game time in athletes or negative career repercussions such as medical disqualification in future pilots (39). Furthermore, perceived costs to physical fitness, military career aspirations, pilot qualifications, sport, reputation, academics, and lack of time in the cadet schedule are emerging as themes as to why USAFA and USMA cadets may choose not to disclose a concussion (40). At USAFA, Mind Matters lessons have been integrated into concussion care and outreach and are annually assessed through school-wide surveys. Mind Matters Challenge–funded investigators and key stakeholders have published consensus recommendations for collegiate athletic departments and Service Academy leaders about how to increase concussion symptom disclosure in their unique settings (41). For a summary of the current Mind Matters Service Academy–specific publications and presentations, see Supplementary Tables 3, 4.

## Academy Specific Aims and Progress

In addition to the work outlined above within the scope of the CARE Consortium CSC and ARC initiatives and the Mind Matters Challenge projects at the service academies, the DoD has leveraged the clinical and research infrastructure in place at these sites to address additional concussion assessment and management objectives with specific relevance to military medical and operational readiness. The purpose of this section is to provide a brief overview of these academy-specific initiatives and provide an update on the progress to date in achieving these objectives.

### Utility of Novel Technologies in the Assessment and Management of Concussion

Several novel technologies have been developed to assist with the clinical assessment and management of concussion. Those technologies have included tools to more objectively quantify postural control (e.g., Tekscan MobileMat™), headsets and software to assess visual system function (e.g., Neurokinetics I-PAS™), and sensors to monitor real-time head impact magnitude and to quantify exposure (e.g., helmet and mouthguard). Currently, the research teams are assessing the validity and diagnostic utility of these devices in the military Service Academy environment. Therefore, a subset of USMA and USAFA cadets enrolled in CARE have been participating in various research projects to understand the utility of these technologies when integrated into the assessment and management of concussion to enhance military operational and medical readiness. The overarching aims are to (1) determine baseline normative values for emerging objective concussion assessment tools and evaluate associations with other baseline assessments and demographic factors, as well as changes in these assessments over time following concussion in injured cases and uninjured controls, and (2) determine cumulative head impact exposure during high-risk Service Academy activities, such as varsity sports and physical education courses, and evaluate associations between head impact exposure, baseline factors, and follow-up concussion assessments in injured cases and uninjured controls.

### Objectively Quantifying the BESS

The BESS has been used for the past two decades to quantify postural control at baseline and post-concussion. During the assessment, a trained human rater visually assesses balance and tallies the number of predefined errors the patient commits during six stances (three positions, on two surfaces). Susceptible to bias and inaccurate scores, as reflected in the BESS test's moderate to good test–retest [Intraclass Correlation Coefficient (ICC) = 0.56–0.75], interrater (ICC = 0.44–0.96), and intrarater (ICC = 0.50–0.98) reliability metrics (20, 42), the traditional human-rated method has faced scrutiny. Tekscan designed the MobileMat™ to improve reliability of the BESS by objectively quantifying errors based on changes in foot pressure. Similarly, the C3 Logix System application utilizes gyroscopes and accelerometers within an Apple iPad to objectively quantify postural sway (iBESS volume) during the BESS assessment. Cadets at USMA have been performing the BESS assessment with an iPad strapped on their back while standing on the MobileMat™, to simultaneously capture the objective outcomes in conjunction with the clinician tallied errors. Since the spring of 2016, more than 400 cadets have completed their baseline BESS assessment on the MobileMat™, and 98 healthy controls have undergone BESS testing on the MobileMat®, in conjunction with the iBESS measuring thoracic postural sway, at three clinically relevant matched time points (i.e., post-injury, return to activity, and 6 months). As a result, reference values have been established for the cadet population, indicating that sex, concussion history, and competitive sport level do not appear to influence BESS performance as measured by the MobileMat™ (43). In the control cohort, level-of-agreement analyses have indicated that the human-rated and MobileMat™ errors tallied may not be comparable, and to ensure consistency, one method should be implemented to measure BESS errors (44). Further analyses are planned to establish the validity of the iBESS and to determine if one system is superior to the other at detecting balance deficits post-concussion.

### Eye Tracking Devices in the Management and Diagnosis of Concussion

Current concussion diagnostic strategies rely heavily on self-reported symptoms as objective diagnostic tools with adequate sensitivity and specificity are lacking. As a result, concussions often go unrecognized or underreported and thus untreated. With a clear need for more objective assessment metrics and a growing body of evidence that concussion can impair eye movement (45–47), several commercially available eye tracking devices have been developed. This process was aided by the Army Directorate of Combat & Doctrine Development Requirements Adjudication Team recognizing the need to develop more objective measures to aid TBI diagnosis in 2014 and, in response, the US Army Medical Research and Materiel Command chartered the Non-Invasive Neuro-Assessment Devices Integrated Product Team (NINAD IPT). For the NINAD IPT to meet its objective of acquiring an operationally effective medical product as quickly and efficiently as possible for use in detection of TBI in active duty service members, product selection criteria were determined as (1) device portability, (2) vendor business plan that includes Food and Drug Administration engagement and review, and (3) sensitivity and specificity for TBI > 85%. As of 2018, there were only three vendors with products mature enough for evaluation: NeuroKinetics, SyncThink, and Oculogica. Researchers at USMA have been exploring the clinical utility of NeuroKinetics' I-Portal Portable Assessment System (I-PAS™). Since May 2018, roughly 300 USMA cadets have completed a baseline I-PAS™ assessment. Preliminary analyses of these data suggest that sex, concussion history, and activity level do not appear to influence the oculomotor and vestibular I-PAS™ assessments (48). Post-concussion assessments have been difficult to capture given the limited portability of the device, as well as, time to set up the device and administer the test battery. To date, USMA has captured I-PAS™ data within 48 h of a concussion for 23 research participants. The data suggest reasonable predictive capabilities for total predicted saccades (49). A saccade is the rapid, simultaneous movement of both eyes from one target to another (50). For saccade testing, targets were displayed at pseudorandom locations along horizontal and vertical axes. The predictive saccade metric was the number of times the cadet accurately predicted the targets. When discriminating between concussed participants and uninjured controls, total predicted saccades had a sensitivity of 75.00%, a specificity of 81.25%, and an area under the receiver operating characteristic curve of 0.81 (49). Additional, data are needed to better understand the utility of these metrics alone and, in combination with other clinical measures, in detecting incident concussions in military service members.

### Head Impact Exposure During High-Risk Activities

In 2007, the Vice Chief of Staff of the Army directed that soldier combat helmets be fitted with electronic sensor technologies. The intent of the sensors was to monitor and record the helmet response to dynamic events, such as exposure to blast events (i.e., improvised explosive devices), ballistic impacts, or blunt impacts. The orthogonal accelerations and blast overpressure levels collected by the sensors could be used to better understand the kinematic and dynamic parameters of operational threats in theater and in turn help define appropriate performance requirements for protective equipment. This order resulted in the creation of the Generation I and Generation II helmet sensors, which have been used to collect head impact data in deployed troops. However, no sensor technologies have been introduced in the non-deployed environment, where at least 80% of TBIs occur (51). Environmental sensors are currently being used at USMA and USAFA to monitor head impact exposure during athletic events. Both academies are using mouthguard and helmet monitors to capture head impact data, such as peak linear and rotational accelerations, head impact location, and season-long risk-weighted exposure, in football and lacrosse athletes participating in the ARC arm of the study. USMA has focused additional efforts on monitoring men's and women's rugby athletes, and USAFA has integrated their mouthguard monitors into club and physical education boxing. Initial findings show that certain subconcussive impacts may affect memory, but more work is needed to explicate this relationship (52). The intent is to utilize the technology to capture head impact exposure, determine impact thresholds that may require sideline evaluation, and to utilize the information to coach athletes on safer playing techniques. The data being collected may also provide information on subclinical head impact exposure during athletic and military training activities in this population. This exploratory data collection will ultimately contribute to continued efforts toward the end goal of developing fieldable technologies that can be used in combat and military training settings to monitor head impact exposure and assess the health consequences of such exposure.

### Academy Specific Biospecimen Collections

A major challenge within the field of concussion injury and management has been to identify objective diagnostic tools for concussion and to monitor recovery from injury. The current standard in concussion assessment and management relies heavily on patient-reported symptoms, although we know there are metabolic and physiologic changes that occur following concussion and that these changes may extend beyond symptom resolution (53, 54). While advances have been made in understanding these metabolic changes post-injury, there are still no clinical tools available with adequate sensitivity and specificity to be used for diagnosis or prognosis following injury, or to monitor recovery and make informed return-to-activity or duty decisions following injury. Other fields such as emergency medicine and cardiology are much more advanced in this area, where validated blood biomarkers, such as troponin, are routinely used to clinically evaluate cardiac damage upon arrival at the emergency room (55). Prior to becoming an ARC site, USMA had launched an investigation to characterize the early biological changes following acute concussion in hopes of identifying promising biomarkers for further study and development. What follows is a brief summary of the non-ARC procedures being carried out at USMA, in conjunction with the CARE Consortium work, to evaluate changes in biomarkers following concussion and their relationship with diagnostic clinical tools and clinical markers of recovery.

During baseline testing, USMA cadets who agreed to participate in the CARE Consortium were also given the opportunity to consent to a separate protocol involving biospecimen collections post-injury. Participants who consented to the local protocol agreed to provide a blood sample, in addition to the clinical measures obtained post-injury as part of CARE, at three clinically relevant time points: within 48 h of injury, return to full duty/activity, and 6 months post-injury. Uninjured control subjects who also consented to the blood biomarkers protocol and matched for age, sex, height, and weight with an injured participant were also identified and provided samples at the corresponding time points. Non-fasting blood samples are collected using standard venipuncture protocol. A total of 22.5 mL of blood is collected using a 10-mL red-top serum tube, a 10-mL K2 EDTA plasma tube, and a 2.5-mL PAXgene RNA tube. In brief, the serum and plasma samples remain on the bench top at room temperature for 30–60 min after collection prior to processing. The serum and plasma samples are centrifuged and aliquoted into cryovials; the first 10 samples are 175 μL in volume, and the remainder of the sample is 500 μL per aliquot. After collection, the plasma sample is also separated for isolation of peripheral blood mononuclear cells (PBMCs) as described elsewhere (56). PBMCs are placed into a slow-freezing container and then transferred to long-term storage, and the RNA tube is processed according to manufacturer guidelines. The PBMCs and RNA will be used for biomarker discovery. The intent is to identify novel biomarkers for concussion using a panomics approach.

To date, USMA has collected biological specimens from 134 concussed cadets and 115 uninjured controls. By time point, USMA has 134 injured and 115 matched-control within-48-h samples, 97 injured and 103 matched-control return-to-full duty/activity samples, and 81 injured and 90 matched-control 6-month samples, and follow-up in this cohort is ongoing. The 620 non-ARC blood collections performed to date have yielded more than 17,000 serum, plasma, and PBMC cryovials. The samples are stored locally at the USMA for future analyses. The plan is to compare the samples between groups, as well as examine the effects over time in injured cadets and uninjured controls. A subset of samples is currently being analyzed to assess differential gene expression in response to concussion and to evaluate the relationships between neurobiological markers of injury and standardized clinical measures of symptomology and performance-based testing (cognitive function, postural stability) immediately post-concussion.

## Developing and Implementing Care at the Service Academies

Developing and implementing a study of this magnitude at any institution, but specifically within the unique Service Academy environment, required extensive planning and coordination. Each site has encountered challenges and learned important lessons that contributed to the overall success of these initiatives at the service academies. The lessons learned through this process have utility in developing and implementing concussion baseline testing in large, at-risk populations at other sites, as well as the potential to inform other institutions engaging in large-scale consortium research.

To ensure success, each academy had to first establish leadership momentum, as initiatives lacking command attention generally receive inadequate resources that can complicate the execution of research projects. As a result, it was imperative to obtain military, academic, athletic, and medical leadership concurrence, as well as permission to implement concussion research within the current model of on-site concussion clinical care. Academy leadership approval was essential to put the complicated research mechanism in place to execute mass baseline testing, including designating time in the Service Academy cadet's and midshipman's busy schedules for a 1.5-h baseline concussion assessment, without interfering with the Academy's primary mission. This extreme sensitivity to the time constraints on cadets and midshipmen had a goal of being time neutral for the respective academies. The medical chain of command support for this project was also essential to scheduling baseline assessments for competitive sports teams and collecting clinically relevant, post-concussion data. Next, each academy had to develop local interdisciplinary teams, composed of key stakeholders (i.e., health care providers, professors, plans analysts, compliance coordinators), and hire research personnel, to assist with developing an implementation strategy to carry out the research. Strategic decisions were made about how the research would be integrated with the current standard of clinical care for concussion, and logistical barriers and solutions were identified. For example, within each site there are various health care providers (i.e., physicians, athletic trainers, physical therapists, nurses, physician assistants) working with participants post-concussion. Thus, setting up a system in which efforts were not duplicated by the research staff was essential to avoiding repetitive testing. As a solution, each academy hired two to four full-time concussion research coordinators to assist the clinical staff and manage the day-to-day data entry. As a result, no additional burden was placed on the clinical providers to implement this large-scale research study.

Other barriers not initially identified have been recognized and resolved throughout the lifespan of the project. As the CARE dataset was designed initially, subject data entry was to be on paper questionnaires requiring subsequent manual input. As a solution to this, all subject response data were input directly via electronic portal entry. This saved significant time for both subjects and staff, and as a result, this solution was utilized across all 30 performance sites. Also, despite educational strategies and support materials to train health care providers, the operational definition for the post-injury time point initially referenced as *asymptomatic* was challenging to interpret and implement consistently based on various guidelines in the literature (35, 57). As a result, it was decided that the *asymptomatic* data collection would occur when the clinician cleared the patient to begin a graduated return to activity protocol, as opposed to when the patient reported zero symptoms. Overall, successful implementation of baseline testing for a large population or executing a large multisite study requires consistent administrative support, a reliable team, and flexible management of the entire research team so that barriers can be identified and resolved.

## Future Research

### Integration of CARE and SALTOS

CARE 2.0 efforts will close out in 2020; however, the service academies, in combination with the Uniformed Services University of Health Sciences, are committed to continuing to work with the CARE Consortium. Specifically, the academies will continue to execute the SALTOS. SALTOS will leverage existing CARE Consortium research efforts and infrastructure at the Service Academy sites to follow cadets and midshipmen up to and following graduation to assess longer-term outcomes following concussion. In addition to the CARE outcome measures, SALTOS includes outcomes that pertain to military performance, overall health, and health care utilization, as well as biological and physiological profiles that may provide prognostic value. The CARE and SALTOS infrastructure and the participants enrolled in these initiatives provide a unique opportunity for an in-depth prospective longitudinal follow-up study of the long-term effects of concussion and repetitive head impact exposure on warfighter and civilian brain health. Funding for such an initiative has been applied for and is under review.

### Concussion, Musculoskeletal Injury Risk, and CARE

Preliminary cross-sectional studies suggest that individuals are at increased risk of lower extremity musculoskeletal injury following concussion (58–60). While several studies have shown an association between concussion and lower extremity injury, many of those investigations have important methodological concerns and limitations. Investigators at USMA intend to utilize the framework of the CARE Consortium in conjunction with local injury surveillance systems to prospectively investigate risk factors for both upper and lower extremity musculoskeletal injury following concussion in cadets. The overarching goal is to identify risk factors associated with musculoskeletal injury following concussion so that strategies can be implemented into concussion protocols to reduce the risk of further injury. The investigation will address many of the notable gaps in the existing literature and will prospectively examine the risk of musculoskeletal injury following concussion to understand the association between the acute deficits following concussion (i.e., balance, cognitive), recovery timelines, and sex on musculoskeletal injury risk during the first 12 months of unrestricted activity post-concussion. If successful, these efforts may be expanded to explore these trends at the other service academies. Better understanding of these factors may inform concussion management and the primary prevention of musculoskeletal injury in patients returning to physically demanding occupations, athletics, or military service.

### Panomics Approach to Biomarker Discovery and Validation

**Figure 3:**
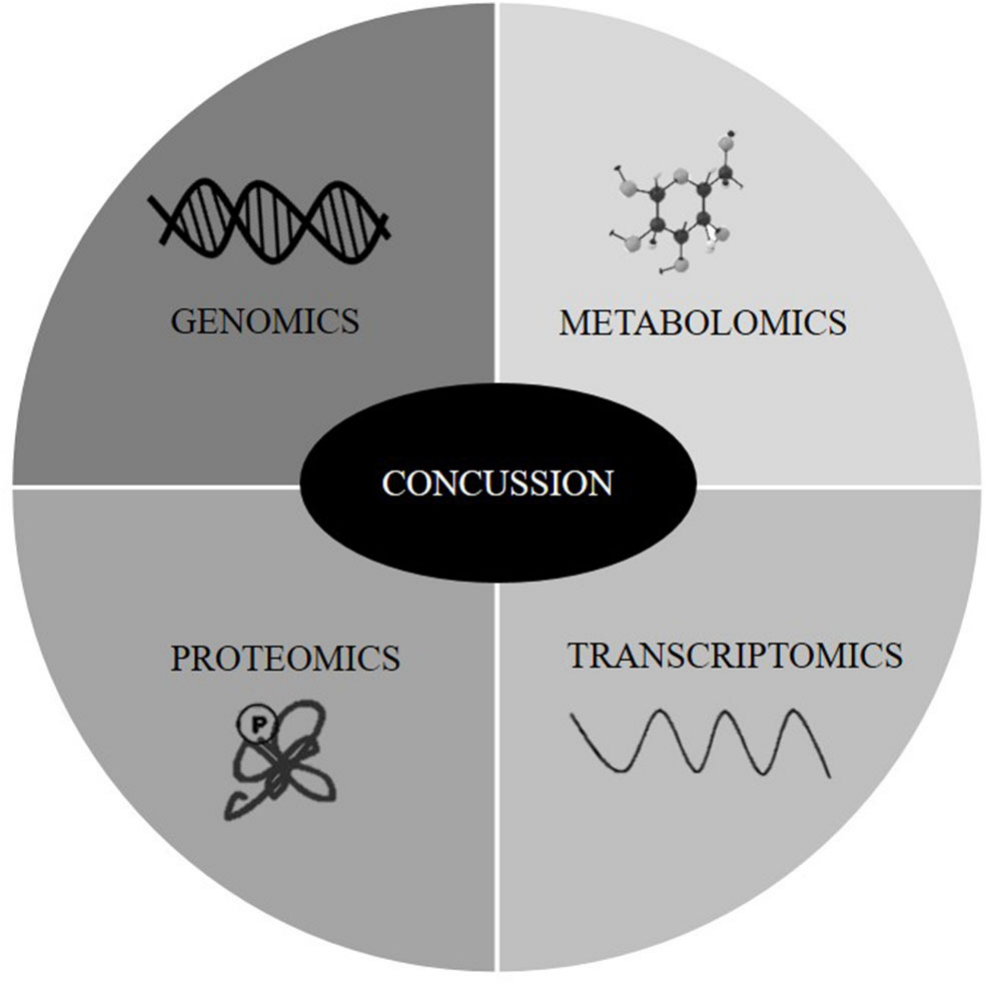
Panomics approach to understanding clinical outcomes associated with concussion alongside functional outcomes.

Individual biomarker analyses have produced promising findings (53), but the current state of concussion biomarkers has not yet been translated to clinical practice. In order to address the complex pathways associated with concussion injury, recovery, and long-term outcomes, studies on the use of parallel system-wide approaches to characterize the biological changes that occur post-injury alongside the functional outcomes are warranted. The goal of such analyses should be to identify candidate biomarkers that (1) can provide prognostic information related to injury risk prediction, (2) have adequate sensitivity and specificity for diagnostic purposes, and (3) can be used to monitor recovery or disease progression. The intersection of the fields of genomics, transcriptomics, proteomics, and metabolomics has the potential to provide a panomics approach to understanding the clinical outcomes of concussion (Figure 3). However, these emerging fields are limited in scope and breadth in nascent concussion research (61). Genomics provides foundational knowledge about a subject's underlying genetic variation that may result from copy number variation or single-nucleotide polymorphisms, whereas transcriptomics and proteomics provide gene expression information. Taken together, comprehensive genomic, transcriptomic, and proteomic data should fundamentally reflect a logical sequence of events from gene to transcript to protein, inclusive of many regulatory features such as splicing, protein turnover, and even post-translational modification of proteins. Metabolomics data, on the other hand, while being generally derived from a complex suite of expressed genes, may also reflect external influences such as diet and injury and thus provide complementary information for other -omics datasets. The combination of analyses using these independent and layered techniques provides a total picture of a subject's complex biological networks responding to an injury and evolving over time to characterize biological responses to injury and recovery, or lack thereof.

The research team at USMA has already begun analyzing the non-ARC samples discussed previously in collaboration with investigators at the National Institutes of Health. The primary objective of the first project is to determine the levels of a panel of known brain injury protein biomarkers in plasma, as well as to perform transcriptomic profiling of the PBMCs of concussed participants compared to age-, sex-, and height-matched uninjured controls. The secondary objective is to examine the overlap between molecular biomarker concentrations and clinical outcomes of recovery. Long-term follow-up in this cohort, possibly leveraging the US DoD Serum Repository and Joint Pathology Center, will be pursued to better understand how concussion impacts military readiness and the clinical outcomes of the warfighter over the course of their military career. This outcome-based research will focus on establishing a longitudinal panomic-derived diagnostic fingerprint (i.e., genome, transcriptome, proteome, and metabolome) with intent of determining whether it is possible to detect or predict long-term neurodegeneration, such as in the case of chronic traumatic encephalopathy. The panomic fingerprint may also allow for the determination of possible therapeutic targets, with the goal to eliminate or slow the progress of neurological diseases associated with concussion.

## Conclusions

In summary, the CARE Consortium work and related academy-specific initiative at the US service academies have built the foundation and established the infrastructure for studying both the short- and long-term effects of concussion in US military service members. The CARE study in conjunction with the individual research endeavors being conducted at each US Service Academy will lead to scientific discoveries that will improve the sensitivity and specificity of current concussion diagnostic tools, optimizing treatment strategies, and prevent or slow the development of neurodegenerative diseases associated with neurotrauma. Although a large portion of the data has yet to be analyzed, the large scope of these concussion assessment and management initiatives will allow for greater generalizability of the findings. The work being conducted at the service academies ultimately intends to improve our knowledge of the natural history of concussion so that we can improve care, increase military readiness, and reduce the burden on service members who become injured.

## Author Contributions

MH, KO'D, JT, RB, JW, and KC contributed to manuscript development and writing. All authors contributed to manuscript revision and read and approved the final version.

## Conflict of Interest

The authors declare that the research was conducted in the absence of any commercial or financial relationships that could be construed as a potential conflict of interest.

## References

[1] Dewan MC Rattani A Gupta S Baticulon RE Hung YC Punchak M. Estimating the global incidence of traumatic brain injury. J Neurosurg. (2018) 130:1080–97. 10.3171/2017.10.JNS17352 29701556

[2] Carney N Ghajar J Jagoda A Bedrick S Davis-O'Reilly C du Coudray H Concussion guidelines step 1: systematic review of prevalent indicators. Neurosurgery. (2014) 75(Suppl. 1):S3–15. 10.1227/NEU.0000000000000433 25006974

[3] McCrory P Meeuwisse WH Aubry M Cantu B Dvorak J Echemendia RJ. Consensus statement on concussion in sport: the 4th international conference on concussion in sport, zurich, november 2012. J Athl Train. (2013) 48:554–75. 10.1097/JSM.0b013e31828b67cf 23855364 PMC3715021

[4] Huber BR Alosco ML Stein TD McKee AC. Potential long-term consequences of concussive and subconcussive injury. Phys Med Rehabil Clin N Am. (2016) 27:503–11. 10.1016/j.pmr.2015.12.007 27154859 PMC4866819

[5] McAllister T McCrea M. Long-term cognitive and neuropsychiatric consequences of repetitive concussion and head-impact exposure. J Athl Train. (2017) 52:309–17. 10.4085/1062-6050-52.1.14 28387556 PMC5384827

[6] Casper ST. Concussion: a history of science and medicine, 1870-2005. Headache. (2018) 58:795–810. 10.1111/head.13288 29536502

[7] Erichsen JE On railway and other injuries of the nervous system. Br Foreign Med Chir Rev. (1867) 40:102–9. PMC5171749 30163223

[8] Page HW A clinical lecture on some cases of head-injury, including one in which there was lesion of the occipital lobe. Lancet. (1901) 157:156–8. 10.1016/S0140-6736(01)81227-7

[9] Casper ST The Neurologists: A History of a Medical Specialty in Modern Britain. 1789–2000. Manchester: Manchester University Press (2014). 10.7228/manchester/9780719091926.001.0001

[10] Tanielian T Jaycox L Invisible Wounds of War: Psychological and Cognitive Injuries, Their Consequences, and Services to Assist Recovery: Rand Corporation. Santa Monica, CA: RAND Corporation (2008). 10.1037/e527612010-001

[11] Defense and Veterans Brain Injury Center DoD Numbers for Traumatic Brain Injury Worldwide-Totals. Available online at: http://dvbic.dcoe.mil/files/tbi-numbers/WorldwideTotals2000-2017Q1-Q3Nov%2014-2017508.pdf (accessed February 10, 2020).

[12] Cameron KL Marshall SW Sturdivant RX Lincoln AE. Trends in the incidence of physician-diagnosed mild traumatic brain injury among active duty US military personnel between 1997 and 2007. J Neurotrauma. (2012) 29:1313–21. 10.1089/neu.2011.2168 22332633

[13] McKee AC Robinson ME. Military-related traumatic brain injury and neurodegeneration. Alzheimers Dement. (2014) 10(Suppl. 3):S242–53. 10.1016/j.jalz.2014.04.003 24924675 PMC4255273

[14] Bryant R. Post-traumatic stress disorder vs traumatic brain injury. Dialogues Clin Neurosci. (2011) 13:251–62. 22034252 10.31887/DCNS.2011.13.2/rbryant PMC3182010

[15] Broglio SP McCrea M McAllister T Harezlak J Katz B Hack D. A national study on the effects of concussion in collegiate athletes and US military service academy members: the NCAA-DoD Concussion Assessment, Research and Education (CARE) consortium structure and methods. Sports Med. (2017) 47:1437–51. 10.1007/s40279-017-0707-1 28281095 PMC5488134

[16] Broglio SP Katz BP Zhao S McCrea M McAllister T Test-retest reliability and interpretation of common concussion assessment tools: findings from the NCAA-DoD care consortium. Sports Med. (2018) 48:1255–68. 10.1007/s40279-017-0813-0 29138991 PMC5889766

[17] Schatz P Sandel N. Sensitivity and specificity of the online version of ImPACT in high school and collegiate athletes. Am J Sports Med. (2013) 41:321–6. 10.1177/0363546512466038 23144368

[18] Vincent AS Roebuck-Spencer TM Cox-Fuenzalida LE Block C Scott JG Kane R. Validation of ANAM for cognitive screening in a mixed clinical sample. Appl Neuropsychol Adult. (2018) 25:366–75. 10.1080/23279095.2017.1314967 28448160

[19] Barr WB McCrea M. Sensitivity and specificity of standardized neurocognitive testing immediately following sports concussion. J Int Neuropsychol Soc. (2001) 7:693–702. 10.1017/S1355617701766052 11575591

[20] Bell DR Guskiewicz KM Clark MA Padua DA. Systematic review of the balance error scoring system. Sports Health. (2011) 3:287–95. 10.1177/1941738111403122 23016020 PMC3445164

[21] Meachen SJ Hanks RA Millis SR Rapport LJ. The reliability and validity of the brief symptom inventory-18 in persons with traumatic brain injury. Arch Phys Med Rehabil. (2008) 89:958–65. 10.1016/j.apmr.2007.12.028 18452746

[22] King PR Donnelly KT Donnelly JP Dunnam M Warner G Kittleson CJ. Psychometric study of the neurobehavioral symptom inventory. J Rehabil Res Dev. (2012) 49:879–88. 10.1682/JRRD.2011.03.0051 23299259

[23] Green RE Melo B Christensen B Ngo LA Monette G Bradbury C. Measuring premorbid IQ in traumatic brain injury: an examination of the validity of the Wechsler Test of Adult Reading (WTAR). J Clin Exp Neuropsychol. (2008) 30:163–72. 10.1080/13803390701300524 18213530

[24] Blevins CA Weathers FW Davis MT Witte TK Domino JL. The posttraumatic stress disorder checklist for DSM-5 (PCL-5): development and initial psychometric evaluation. J Trauma Stress. (2015) 28:489–98. 10.1002/jts.22059 26606250

[25] Chin EY Nelson LD Barr WB McCrory P McCrea MA. Reliability and validity of the Sport Concussion Assessment Tool-3 (SCAT3) in high school and collegiate athletes. Am J Sports Med. (2016) 44:2276–85. 10.1177/0363546516648141 27281276

[26] Bacon DR Bean B GPA in research studies: an invaluable but neglected opportunity. J Mark Educ. (2006) 28:35–42. 10.1177/0273475305284638

[27] Knapik J. The Army Physical Fitness Test (APFT): a review of the literature. Mil Med. (1989) 154:326–9. 10.1093/milmed/154.6.326 2498771

[28] O'Connor KL Dain Allred C Cameron KL Campbell DE D'Lauro CJ Houston MN. Descriptive analysis of a baseline concussion battery among US. service academy members: results from the Concussion Assessment, Research, and Education (CARE) Consortium. Mil Med. (2018) 183:e580–90. 10.1093/milmed/usx130 29608767

[29] Roach SP Houston MN Peck KY Svoboda CSJ Kelly TF Malvasi SR. The influence of self-reported tobacco use on baseline concussion assessments. Mil Med. (2019) 185:e431–7. 10.1093/milmed/usz352 31603220

[30] Caccese JB Iverson GL Cameron KL Houston MN McGinty GT Jackson JC. Estimated age of first exposure to contact sports is not associated with greater symptoms or worse cognitive functioning in male US service academy athletes. J Neurotrauma. (2020) 37:334–9. 10.1089/neu.2019.6571 31375052 PMC7364303

[31] Bookbinder HA Houston MN Peck KY Habecker S Colsant BJ Kelly TF. Factors associated with delayed concussion reporting in military academy cadets. J Athl Train. (2020) 55:843–9. 10.4085/1062-6050-362-19 32607554 PMC7462178

[32] Van Pelt KL Allred D Cameron KL Campbell DE D'Lauro CJ He X. A cohort study to identify and evaluate concussion risk factors across multiple injury settings: findings from the CARE consortium. Inj Epidemiol. (2019) 6:1. 10.1186/s40621-018-0178-3 30637568 PMC6330552

[33] Van Pelt KL Allred D Brodeur R Cameron KL Campbell DE D'Lauro CJ. Concussion recovery trajectories among tactical athletes: results from the CARE consortium. J Athl Train. (2020) 55:658–65. 10.4085/1062-6050-10-19 32556201 PMC7384467

[34] D'Lauro C Johnson BR McGinty G Allred CD Campbell DE Jackson JC. Reconsidering return-to-play times: a broader perspective on concussion recovery. Orthop J Sports Med. (2018) 6:2325967118760854. 10.1177/2325967118760854 29568786 PMC5858632

[35] McCrory P Meeuwisse W Dvorak J Aubry M Bailes J Broglio S. Consensus statement on concussion in sport—the 5th international conference on concussion in sport held in Berlin, october 2016. Br J Sports Med. (2017) 51:838–47. 10.1136/bjsports-2017-097699 28446457

[36] Register-Mihalik JK Cameron KL Kay MC Kerr ZY Peck KY Houston MN. Determinants of intention to disclose concussion symptoms in a population of US. Military cadets. J Sci Med Sport. (2019) 22:509–15. 10.1016/j.jsams.2018.11.003 30551922

[37] Register-Mihalik JK Kay MC Kerr ZY Peck KY Houston MN Gildner P. Influence of concussion education exposure on concussion-related educational targets and self-reported concussion disclosure among first-year service academy cadets. Mil Med. (2019) 185:e403–9. 10.1093/milmed/usz414 31789379

[38] Johnson BR McGinty GT Jackson JC Ramsey TM Hjalber M Hill C. Return-to-learn: a post-concussion academic recovery program at the US. Air force academy. Mil Med. (2018) 183:101–4. 10.1093/milmed/usx106 29547933

[39] Foster CA D'Lauro C Johnson BR. Pilots and athletes: different concerns, similar concussion non-disclosure. PLoS ONE. (2019) 14:e0215030. 10.1371/journal.pone.0215030 31042725 PMC6493720

[40] Rawlins MLW Johnson BR Register-Mihalik JK DeAngelis K Schmidt JD D'Lauro CJ. United States Air Force academy cadets' perceived costs of concussion disclosure. Mil Med. (2020) 185:e269–75. 10.1093/milmed/usz162 31268525

[41] Kroshus E Cameron KL Coatsworth JD D'Lauro C Kim NJ Lee KM. Actionable approaches to improving concussion care seeking: consensus from the NCAA-department of defense mind matters research and education grand challenge. Br J Sports Med. (2020). 10.1136/bjsports-2020-102185. [Epub ahead of print]. 32912847

[42] Barlow M Schlabach D Peiffer J Cook C. Differences in change scores and the predictive validity of three commonly used measures following concussion in the middle school and high school aged population. Int J Sports Phys Ther. (2011) 6:150–7. 21904694 PMC3163995

[43] Houston MN Peck KY Malvasi SR Roach SP Svoboda SJ Cameron KL. Reference values for the balance error scoring system as measured by the tekscan mobilemat in a physically active population. Brain Inj. (2019) 33:299–304. 10.1080/02699052.2018.1552021 30501390

[44] Houston MN Hoch MC Malvasi SR Peck KY Svoboda SJ Cameron KL. Level of agreement between human-rated and instrumented balance error scoring system scores. Ann Biomed Eng. (2019) 47:2128–35. 10.1007/s10439-019-02274-5 31011917

[45] Wright WG Tierney RT McDevitt J. Visual-vestibular processing deficits in mild traumatic brain injury. J Vestib Res. (2017) 27:27–37. 10.3233/VES-170607 28387693

[46] Cifu DX Wares JR Hoke KW Wetzel PA Gitchel G Carne W. Differential eye movements in mild traumatic brain injury versus normal controls. J Head Trauma Rehabil. (2015) 30:21–8. 10.1097/HTR.0000000000000036 24695263

[47] Kelly KM Kiderman A Akhavan S Quigley MR Snell ED Happ E. Oculomotor, vestibular, and reaction time effects of sports-related concussion: video-oculography in assessing sports-related concussion. J Head Trauma Rehabil. (2019) 34:176–88. 10.1097/HTR.0000000000000437 30234848 PMC6553977

[48] Roach SP Houston MN Peck KY Malvasi SR Cameron KL Reference values for oculomotor and vestibular outcomes in military cadets. In: Eastern Athletic Trainers' Association Meeting & Clinical Symposium. Valley Forge, PA (2019).

[49] Houston MN King J Zhang D Ross JD Malvasi SR Cameron KL Ability of an eye-tracking device for detecting concussion in military cadets: a pilot study. In: Journal of Athletic Training. National Athletic Trainers' Association Meeting & Clinical Symposium. Atlanta (2020).

[50] Termsarasab P Thammongkolchai T Rucker JC Frucht SJ. The diagnostic value of saccades in movement disorder patients: a practical guide and review. J Clin Mov Disord. (2015) 2:14. 10.1186/s40734-015-0025-4 26788350 PMC4710978

[51] Centers for Disease Control and Prevention Report to Congress on Traumatic Brain Injury in the United States: Epidemiology and Rehabilitation. National Center for Injury Prevention and Control (2015). p. 1–72. Available online at: https://www.cdc.gov/traumaticbraininjury/pdf/TBI_Report_to_Congress_Epi_and_Rehab-a.pdf 10.1016/j.apmr.2015.07.001 26184889

[52] Leeds DD D'Lauro C Johnson BR. Predictive power of head impact intensity measures for recognition memory performance. Mil Med. (2019) 184(Suppl. 1):206–17. 10.1093/milmed/usy415 30901472

[53] McCrea M Broglio SP McAllister TW Gill J Giza CC Huber DL. Association of blood biomarkers with acute sport-related concussion in collegiate athletes: findings from the NCAA and department of defense CARE consortium. JAMA Netw Open. (2020) 3:e1919771. 10.1001/jamanetworkopen.2019.19771 31977061 PMC6991302

[54] Giza CC Hovda DA. The new neurometabolic cascade of concussion. Neurosurgery. (2014) 75:S24–33. 10.1227/NEU.0000000000000505 25232881 PMC4479139

[55] Body R Hendry C Cardiac biomarkers in emergency care. Cardiol Clin. (2018) 36:27–36. 10.1016/j.ccl.2017.09.002 29173679

[56] Green H Zhang X Tiklova K Volakakis N Brodin L Berg L. Alterations of p11 in brain tissue and peripheral blood leukocytes in Parkinson's disease. Proc Natl Acad Sci USA. (2017) 114:2735–40. 10.1073/pnas.1621218114 28137881 PMC5347629

[57] Broglio SP Cantu RC Gioia GA Guskiewicz KM Kutcher J Palm M. National athletic trainers' association position statement: management of sport concussion. J Athl Train. (2014) 49:245–65. 10.4085/1062-6050-49.1.07 24601910 PMC3975780

[58] Lynall RC Mauntel TC Padua DA Mihalik JP. Acute lower extremity injury rates increase after concussion in college athletes. Med Sci Sports Exerc. (2015) 47:2487–92. 10.1249/MSS.0000000000000716 26057941

[59] Gilbert FC Burdette GT Joyner AB Llewellyn TA Buckley TA. Association between concussion and lower extremity injuries in collegiate athletes. Sports Health. (2016) 8:561–7. 10.1177/1941738116666509 27587598 PMC5089357

[60] Houston MN Hoch JM Cameron KL Abt JP Peck KY Hoch MC. Sex and number of concussions influence the association between concussion and musculoskeletal injury history in collegiate athletes. Brain Inj. (2018) 32:1353–8. 10.1080/02699052.2018.1512718 30136896

[61] Sandhu C Qureshi A Emili A. Panomics for precision medicine. Trends Mol Med. (2018) 24:85–101. 10.1016/j.molmed.2017.11.001 29217119 PMC5839674

