# Supplementary material for: Progress and Future Directions of the NCAA-DoD Concussion Assessment, Research, and Education (CARE) Consortium and Mind Matters Challenge at the US Service Academies
Source: Front Neurol. 2020 Sep 24;11:542733. doi: 10.3389/fneur.2020.542733 (PMC7546354; doi:10.3389/fneur.2020.542733)
Supplement: Supplementary file 1 [file Table_1.docx]

**Table S1. Summary of Service Academy-Specific CARE Manuscripts**

| **Study** | **Year** | **Title** | **Journal** | **Main Findings** |
| --- | --- | --- | --- | --- |
| Bookbinder et al | 2020 | Factors associated with delayed concussion reporting in military academy cadets | *J Athl Train* | Roughly half (51%) of cadets that sustain a concussion fail to immediately report their injury. The dataset suggest that females, cadets injured outside of competition, and highly competitive athletes are almost twice as likely to delay reporting. |
| Caccese et al | 2020 | Estimated age of first exposure to contact sports is not associated with greater symptoms or worse cognitive functioning in male U.S. Service Academy athletes | *J Neurotrauma* | Earlier exposure to contact sports was not associated with worse neurocognitive test performance or greater symptom reporting at baseline. |
| Houston et al | 2020 | Test-Retest Reliability of Concussion Baseline Assessments in United States Service Academy Cadets: A Report from the NCAA-DoD CARE Consortium | *J Int Neuropsychol Soc* | ICC values for the 1-year test interval ranged from 0.28-0.67 and from 0.15-0.57 for the 2-year interval. Kappa values ranged from 0.16-0.21 for the 1-year interval and from 0.29-0.31 for the 2-year test interval. Across all measures, the observed effects were small, ranging from 0.01-0.44. This investigation noted less than optimal reliability for the most common concussion baseline assessments. While none of the assessments met or exceeded the accepted clinical threshold, the effect sizes were relatively small suggesting an overlap in performance from year-to-year. |
| Van Pelt et al | 2020 | Concussion recovery trajectories among tactical athletes: Results from the CARE Consortium. | *J Athl Train* | Cadets who reported >11 symptoms on the Sport Concussion Assessment Tool (SCAT) within 48 hours of concussion had 1.59 times longer symptom recovery duration (95% CI: 1.05-2.39) compared to those with ≤11 symptoms. Varsity athlete cadets took less time than non-varsity cadets to become asymptomatic (HR: 9.19 95% CI: 5.40-15.64). Similarly to symptom duration, having >11 symptoms on the SCAT was associated with longer return to activity (RTA) protocol durations (HR: 0.77 95% CI: 0.65-0.90) and varsity status was associated with shorter RTA protocol duration (HR: 1.76 95% CI: 1.36-2.29). |
| *Houston et al | 2019 | Level of agreement between human-rated and instrumented Balance Error Scoring System scores | *Ann Biomed Eng* | Agreement between human-rated and instrumented BESS scores was poor for all stances and thus may not be comparable. One method should be used to measure BESS errors for consistency. |
| *Houston et al | 2019 | Reference values for the Balance Error Scoring System as measured by the Tekscan MobileMat in a physically active population | *Brain Inj* | Previously reported BESS reference values in collegiate athletes and adolescents were slightly higher than USMA cadets. Sex, concussion history, and competitive sport level do not appear to influence BESS performance as measured by the MobileMat™. |
| Roach et al | 2019 | The influence of self-reported tobacco use on baseline concussion assessments | *Mil Med* | Cadets that used tobacco performed significantly worse than non-tobacco users on the impulse control (*P <*.001, *Cohen’s d* =0.06) section of the ImPACT, reported greater ImPACT symptom severity scores (*p <*.001, *Cohen’s d*=0.06), and were more likely to take risks (*p<.*001, *Cohen’s d*=0.18). No differences were detected for Balance Error Scoring System, Standardized Assessment of Concussion, Brief Symptom Inventory-18, and Sport Concussion Assessment Tool symptom scores, verbal memory, visual memory, visual-motor speed, or reaction time on the ImPACT (*p>*.004). These differences should be interpreted with caution as the effect sizes were very small. |
| Van Pelt et al | 2019 | A cohort study to identify and evaluate concussion risk factors across multiple injury settings: findings from the CARE Consortium | *Inj Epidemiol* | Female sex and previous concussion were the most consistent estimators of concussion risk across all concussion settings. Compared to males, females had 2.02 (95%CI: 1.70–2.40) times the risk of a concussion regardless of injury setting, and greater relative risk when the concussion occurred during sport (Odds Ratio (OR): 1.38 95% CI: 1.07–1.78). Previous concussion was associated with 1.98 (95% CI: 1.65–2.37) times increased risk for any incident concussion, and the magnitude was relatively stable across all concussion settings (OR: 1.73 to 2.01). Freshman status was also associated with increased overall concussion risk, but was driven by increased risk for academy training-related concussions (OR: 8.17 95% CI: 5.87–11.37). Medical history of headaches in the past 3months, diagnosed ADD/ADHD, and BSI-18 Somatization symptoms increased overall concussion risk. |
| D’Lauro et al | 2018 | Reconsidering return-to-play Times: A broader persepctive on concusison recovery | *Orthop J Sports Med* | Overall mean return to play was 29.4 days. Sex and athletic status both affected return-to-play time. Men showed significantly shorter return to play than women, taking 24.7 days versus 35.5 days (*P*<.001). Intercollegiate athletes also reported quicker return-to-play times than non-intercollegiate athletes: 25.4 days versus 34.7 days (*P*=.002) |
| O’Connor et al | 2018 | Descriptive analysis of a baseline concussion battery among U.S. Service Academy members: Results from the Concussion Assessment, Research, and Education (CARE) Consortium | *Mil Med* | This is the first investigation to provide normative baseline data for U.S. Service Academy members. The results highlight the general uniformity of baseline performance across sex, competition level, and contact level at the service academies. |

*Denotes a Department of Defense Academy-Specific initiative leveraged in conjunction with CARE
