# Supplementary material for: Progress and Future Directions of the NCAA-DoD Concussion Assessment, Research, and Education (CARE) Consortium and Mind Matters Challenge at the US Service Academies
Source: Front Neurol. 2020 Sep 24;11:542733. doi: 10.3389/fneur.2020.542733 (PMC7546354; doi:10.3389/fneur.2020.542733)
Supplement: Supplementary file 2 [file Table_2.docx]

**Table S2. List of Service Academy-Specific CARE Abstract Presentations**

| **Study** | **Year** | **Title** | **Presentation Forum** |
| --- | --- | --- | --- |
| Aderman et al | 2020 | Association between symptom burden at initiation of a graduated return to activity protocol and time to return to unrestricted activity after concussion in Service Academy cadets | *National Athletic Trainers’ Association* |
| *Houston et al | 2020 | Ability of an eye-tracking device for detecting concussion in military cadets: A pilot study | *National Athletic Trainers’ Association* |
| *Ross et al | 2020 | The relationship between human-rated errors and tablet-based postural sway during the Balance Error Scoring System in military cadets | *National Athletic Trainers’ Association* |
| Bookbinder et al | 2019 | Test-retest reliability of the BSI-18 in United States Service Academy cadets: Findings from the NCAA-DoD CARE Consortium | *National Athletic Trainers’ Association* |
| Caccese et al | 2019 | Age of first exposure to contact sports and neurocognitive performance in U.S. Service Academy athletes | *Sports Neuropsychology Society Symposium* |
| Giza et al | 2019 | Acute biomarkers in military cadets sustaining concussions outside of varsity sports participation | *Military Health System Research Symposium* |
| *Malvasi et al | 2019 | Headache Impact Test-6 scores pre-to post concussion in Service Academy cadets | *National Athletic Trainers’ Association* |
| *Roach et al | 2019 | Reference values for oculomotor and vestibular outcomes in military cadets | *Eastern Athletic Trainers’ Association* |
| Stemper et al | 2019 | Changes in preseason head impact exposure for football athletes at the military Service Academies following 2017 and 2018 NCAA rule changes | *Military Health System Research Symposium* |
| Cameron et al | 2018 | Risk of concussion by sex and activity in U.S. Service Academy cadets | *American College of Sports Medicine* |
| Houston et al | 2018 | Test-retest reliability of the Immediate Post-Concussion Assessment and Cognitive Test in United States Service Academy Cadets: A report from the Concussion Assessment, Research, and Education Consortium | *National Athletic Trainers’ Association* |
| Jackson et al | 2018 | Concussion and mental health among United States Service Academy cadets | *American College of Sports Medicine* |
| *Malvasi et al | 2018 | Level of agreement between human-rated and computerized Balance Error Scoring System | *National Athletic Trainers’ Association* |
| O’Connor et al | 2018 | Individual symptoms classify typical and atypical concussion recovery duration | *Military Health System Research Symposium* |
| O’Connor et al | 2018 | Concussion recovery trajectories among United States Service Academy members | *American College of Sports Medicine* |
| *Roach et al | 2018 | Reference values for the Headache Impact Test-6 in military cadets | *National Athletic Trainers’ Association* |
| Carminati et al | 2017 | Factors associated with delayed concussion reporting in military academy cadets | *National Athletic Trainers’ Association* |
| *Houston et al | 2017 | Reference values for the Balance Error Scoring System in military cadets | *National Athletic Trainers’ Association* |
| Malvasi et al | 2017 | Association between symptom severity and number of symptoms post-injury and symptom resolution and return to participation following concussion | *National Athletic Trainers’ Association* |
| O’Connor et al | 2017 | Contact sport exposure does not have detrimental effect on baseline neurocognitive performance or symptoms. | *World Congress of Neurology* |
| O’Connor et al | 2017 | Effect of environment on neurocognitive testing | *Military Health System Research Symposium* |
|  |  |  |  |
|  |  |  |  |
| Roach et al | 2017 | The influence of self-reported tobacco use on baseline concussion assessments among Service Academy cadets | *National Athletic Trainers’ Association* |
| O’Connor et al | 2016 | The prevalence of concussion within the military academies: Findings from the Concussion Assessment, Research, and Education (CARE) Consortium. | *Concussion Consensus Meeting* |

*Denotes a Department of Defense academy-specific initiative leveraged in conjunction with CARE
