# Supplementary material for: Progress and Future Directions of the NCAA-DoD Concussion Assessment, Research, and Education (CARE) Consortium and Mind Matters Challenge at the US Service Academies
Source: Front Neurol. 2020 Sep 24;11:542733. doi: 10.3389/fneur.2020.542733 (PMC7546354; doi:10.3389/fneur.2020.542733)
Supplement: Supplementary file 3 [file Table_3.docx]

**Table S3. Summary of Service Academy-Specific Mind Matters Manuscripts**

| **Study** | **Year** | **Title** | **Journal** | **Main Findings** |
| --- | --- | --- | --- | --- |
| Kroshus et al | 2020 | Actionable approaches to improving concussion care seeking: Consensus from the NCAA-Department of Defense Mind Matters Research and Education Grand Challenge | *Br J Sports Med* | Key additions to the content of concussion education should include short-term benefits of early symptom disclosure and the potential dilemma individuals face when deciding to disclose a concussion. Improving concussion disclosure requires more than improving the content of concussion education. This includes attending to how concussion education is disseminated and ensuring that team/unit and organizational processes positively reinforce rather than undermine concussion education messaging. |
| Rawlins et al | 2020 | United States Air Force Academy cadets' perceived costs of concussion disclosure | *Mil Med* | The following subthemes were identified: perceived costs to physical fitness, military career aspirations, pilot qualifications, sport, reputation, academics, and lack of time. By acknowledging and targeting these perceived cost subthemes more cadets with concussions may choose to report an injury, receive treatment sooner, and potentially lessen the long-term impact. |
| Foster et al | 2019 | A social dilemma model of information self-disclosure, applied to the concussion domain | *Journal of Concussion* | Providing an explicit theoretical basis for self-disclosure dilemmas is critical for understanding concussion non-disclosure and mitigating its corresponding harm. This social dilemma model highlights (a) the foundational psychological basis for concussion non-disclosure, (b) possible reasons that initiatives designed to increase concussion disclosure have been ineffective, and (c) the need to consider the decision-making autonomy of concussed individuals. This model may be applicable to any domain where individuals are reluctant to disclose personal information to others who need it. |
| Foster et al | 2019 | Pilots and athletes: Different concerns, similar concussion non-disclosure | *PLoS One* | The results demonstrate that concussion non-disclosure develops when a population perceives disclosure as more costly and less rewarding. *Perceived* *Cost* and *Perceived Reward* variables alone accounted for 50% of the variance in *Anticipated* *Conclusion Disclosure* (*Adjusted R^2^ =* 0.50, *F*(2,2312) = 1,145.31, *p*<.001). *Anticipated Conclusion Disclosure* developed for different reasons within different sub-populations. Cadet intercollegiate athletes reported being primarily concerned that concussion self-disclosure would cause them to miss practice or game time (*t* (736.7)=14.20, *p*<.001, *Cohen’s d* = 0.96). Cadet future pilots reported being primarily concerned that concussion disclosure would have negative U.S. Air Force career repercussions (*t*(1828)=10.25, *p*<.001, *Cohen’s d*=0.50) |
| Leeds et al | 2019 | Predictive power of head impact intensity measures for recognition memory performance | *Mil Med* | Boxers received more head impacts and achieved lower performance in post-exercise memory than non-boxers. For several measures of impact motion, impact intensity appeared to set an upper bound on post-exercise memory performance – stronger impacts led to lower expected memory performance. This trend was most significant when impact intensity was measured through a novel technique, applying principal component analysis to boxer motion. Principal component analysis measures also captured more distinct impact information than seven traditional impact measures also tested. |
| Register-Mihalik et al | 2019 | Determinants of intention to disclose concussion symptoms in a population of U.S. military cadets | *J Sci Med Sport* | Concussion-related perceived social norms, attitudes, and perceived control are associated with intention to disclose. In the multivariable model, a 10% shift towards more favorable perceived social norms (PR=1.28; p<0.001) and attitudes (PR=1.07; p=0.05) about concussion were associated with high intention to disclose concussion symptoms. High perceived control over disclosure was associated with high intention to disclose concussion symptoms (PR=1.39; p=0.08). Organizationally appropriate intervention strategies should be developed. |
| Register-Mihalik et al | 2019 | Influence of concussion education exposure on concussion-related educational targets and self-reported concussion disclosure among first-year Service Academy cadets | *Mil Med* | 71.5% of first-year cadets reported receiving some type of previous concussion education and 23.6% reported a concussion history. Of those reporting previous concussion-related education, 78.0% watched a video, 74.0% talked with a coach about concussion, 62.3% talked with a medical professional, and 8.8% reported other sources of education ranging from anatomy teachers to brochures. Overall, 75.8% reported receiving more than one source of concussion education. Having played a contact sport in high school and having a history of concussion were associated with having multiple concussion education exposures. Being female was associated with lower odds of multiple exposures. Exposure to multiple sources of concussion education was not associated with knowledge, attitudes, perceived norms, or higher intention to disclose concussion symptoms. |
| Johnson et al | 2018 | Return-to-learn: A post-concussion academic recovery program at the U.S. Air Force Academy | *Mil Med* | This commentary outlines the U.S. Air Force Academy’s 5-stage return-to-learn protocol. Those stages include: 1) Cognitive Rest, 2) Trial of Cognitive Activity, 3) Return to Class (maximum modifications), 4) Return to Class (minimum modifications), 5) Return to Class (no modifications). |
