# Supplementary material for: Progress and Future Directions of the NCAA-DoD Concussion Assessment, Research, and Education (CARE) Consortium and Mind Matters Challenge at the US Service Academies
Source: Front Neurol. 2020 Sep 24;11:542733. doi: 10.3389/fneur.2020.542733 (PMC7546354; doi:10.3389/fneur.2020.542733)
Supplement: Supplementary file 4 [file Table_4.docx]

**Table S4. List of Service Academy Specific Mind Matters Abstract Presentations**

| **Study** | **Year** | **Title** | **Presentation Forum** |
| --- | --- | --- | --- |
| Callahan et al | 2020 | Assessing influencers of perceived school-level concussion care and support among collegiate student-athletes | *National Athletic Trainers’ Association* |
| Callahan et al | 2020 | Perceived school-level concussion care and support among collegiate student-athletes: Behavioral determinants and intentions to report | *North American Society for the Psychology of Sport and Physical Activity* |
| Register-Mihalik et al | 2020 | Associations between contact/collision sport participation and key concussion care-seeking behaviors among first-year collegiate student-athletes: The BANK Study | *National Athletic Trainers’ Association* |
| D’Lauro et al | 2019 | Interdisciplinary Efforts to Test and Implement Perceived Cost-Based Concussion Interventions at the U.S. Air Force Academy | *Military Health System Research Symposium* |
| Register-Mihalik et al | 2019 | Association between concussion education sources and concussion disclosure knowledge, attitudes, perceived norms, intentions, and behaviors among first-year Service Academy cadets: Implications for concussion education and training | *Military Health System Research Symposium* |
| Weber-Rawlins et al | 2019 | A qualitative study of Concussion Reporting at the U.S. Air Force Academy | *Military Health System Research Symposium* |
| Register-Mihalik et al | 2018 | Factors associated with intention to disclose concussive symptoms among Service Academy cadets: The BANK study | *American College of Sports Medicine* |
| Gallini et al | 2017 | Predictors of concussion-related knowledge, attitudes, beliefs, and disclosure intention in collegiate freshmen student-athletes | *American Public Health Association* |
| Johnson et al | 2017 | Concussion policy myths and their effect on self-report of concussion | *Society for Neuroscience* |
| Register-Mihalik et al | 2017 | Concussion disclosure knowledge, attitudes, beliefs and behaviors among first year Service Academy cadets: The BANK Study | *National Athletic Trainers’ Association* |
| D’Lauro et al | 2016 | Costs and contexts: Factors affecting self-report of concussion in a military population | *5^th^ International Consensus Conference on Concussion in Sport* |
